# Synchronous low-grade myofibroblastic sarcoma and small cell lung cancer in a patient with situs inversus totalis: a case report and literature review

**DOI:** 10.3389/fonc.2026.1782502

**Published:** 2026-03-13

**Authors:** JiaJin Yang, PeiLi Xiong, XiaoMeng Liu, XiaoLin Wei, HuiLan Kuang

**Affiliations:** 1 Department of Oncology, Fengcheng People’s Hospital, Yichun University, Yichun, Jiangxi, China; 2 Department of Respiratory and Critical Care Medicine, Fengcheng People’s Hospital, Yichun University, Yichun, Jiangxi, China; 3 Department of Pathology, Fengcheng People’s Hospital, Yichun University, Yichun, Jiangxi, China; 4 Department of Head and Neck Surgery, Fengcheng People’s Hospital, Yichun Universityi, Yichun, Jiangx, China; 5 Department of Oncology, Ganzhou Cancer Hospital, Ganzhou, Jiangxi, China

**Keywords:** immunotherapy, low- grade myofibroblastic sarcoma, situs inversus totalis, small cell lung cancer, thoracic radiotherapy

## Abstract

**Background:**

Situs inversus totalis (SIT) is a rare congenital anomaly. Its co-occurrence with multiple primary malignancies is exceedingly uncommon. Low-grade myofibroblastic sarcoma (LGMS) and small cell lung cancer (SCLC) represent two distinct neoplasms with markedly different biological behaviors and treatment sensitivities. To our knowledge, the synchronous presentation of LGMS and SCLC in a patient with SIT has not been previously described.

**Case presentation:**

A 72-year-old man with known SIT presented with respiratory symptoms. Imaging revealed a left hilar mass causing obstructive atelectasis, along with metastatic lymphadenopathy in cervical and inguinal regions. Pathological assessment confirmed synchronous extensive-stage SCLC (ES-SCLC) and cervical LGMS. Given the high chemosensitivity of SCLC, a sequential treatment strategy was implemented. The patient first received two cycles of induction chemoimmunotherapy with etoposide/cisplatin plus tislelizumab, which resulted in resolution of metastatic lymph nodes. This was followed by surgical resection of the residual cervical LGMS and four cycles of consolidation chemoimmunotherapy. Subsequent management included sequential thoracic radiotherapy and maintenance immunotherapy with tislelizumab.

**Conclusion:**

This report presents the first documented case of synchronous LGMS and SCLC in a patient with SIT. Our experience suggests that a biology-driven sequential approach, prioritizing systemic therapy for chemosensitive SCLC followed by local control of LGMS, can achieve a initial favorable response. It also underscores that radiotherapy remains feasible in SIT following detailed anatomical evaluation. This case offers a clinically applicable, biology-guided sequential strategy for managing synchronous malignancies with divergent treatment sensitivities.

## 1 Introduction

Situs inversus totalis (SIT) is a rare congenital anomaly marked by a complete mirror-image reversal of thoracic and abdominal organ positions (1), Its etiology may involve defects linked to the X chromosome (2). Reported incidence rates vary across studies, with most estimates ranging between 1 in 6,000 to 1 in 25,000 live births (3, 4). This uncommon condition is typically identified incidentally through thoracic or abdominal imaging (5). SIT itself is not a premalignant condition. However, An association between SIT and multiple malignancies has been documented in the literature, including lung cancer (6), thyroid carcinoma (7), and abdominopelvic cancer (8), among others. A definitive causal link between SIT and an increased risk of malignancy remains unestablished (9).

Low-grade myofibroblastic sarcoma (LGMS) is an uncommon malignant soft-tissue tumor derived from stromal cells. It is defined histologically by the presence of atypical myofibroblasts exhibiting fibromatosis-like features (10, 11), and shows a particular predilection for the head and neck region in adult male with an average age of 40 years (12). According to the 2020 World Health Organization (WHO) classification update for fibroblastic and myofibroblastic tumors, LGMS is recognized as a distinct clinico-pathologic entity categorized as an intermediate-grade, rarely metastasizing neoplasm, thereby clearly differentiating it from both benign and malignant counterparts (13, 14). LGMS represents approximately 0.6% of all soft tissue malignancies (15, 16). Although locally aggressive, it generally carries a more favorable prognosis compared to many other malignant tumors (e.g., small cell lung cancer), characterized by a low metastatic potential and a propensity for local recurrence (17, 18).

In China, lung cancer remains the predominant malignancy and the leading cause of cancer death worldwide (19, 20). Approximately 15% of lung cancers are classified as small cell lung cancer (SCLC) (21), a highly aggressive neuroendocrine tumor known for its rapid growth kinetics, early metastatic potential, and unfavorable clinical outcomes (22). Among all lung cancer subtypes, SCLC exhibits the most significant epidemiological association with intense active or passive smoking (23, 24). Paternal smoking has been identified as a significant risk factor for SIT in a population-based Baltimore-Washington Infant Study (1). Although the risk factors for low-grade malignant myofibroblastic sarcoma remain unknown, it remains unclear whether the external smoking environment is the cause of the synchronous occurrence of small cell lung cancer and low-grade malignant myofibroblastic sarcoma in SIT.

The literature on patients with SIT who have synchronous and metachronous multiple primary malignancies is extremely rare [e.g., lung adenocarcinoma and a pleural-based solitary fibrous tumor (5), Double primary colon cancers (25), double primary lung cancers (26), esophageal carcinoma and lung cancer (27), gastric cancer and gastrointestinal stromal tumor (28)]. No prior reports exist, to our knowledge, describing the co-occurrence of LGMS and SCLC in the setting of SIT. This report illustrates a unique case of a patient who had synchronous LGMS and SCLC with SIT and was treated by Local tumor resection, immunotherapy combined with chemotherapy, and thoracic radiotherapy. Depending on the different heterogeneity and malignancy of the tumors, we also discussed the treatment sequence for synchronous multiple primary tumors.

## 2 Case presentation

### 2.1 Chief complaints

**Figure 1:**
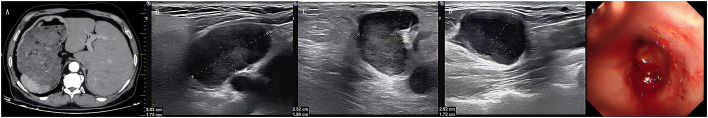
Imaging examination results. **(a)** Enhanced CT shows SIT; ultrasound examination revealed lymph node metastasis of SCLC in the left neck **(b)** and left inguinal region **(c)**; **(d)** ultrasound examination revealed LGMS in zone III of the left neck; **(e)** Bronchoscopy show a neoplasm at the left lower lobe bronchial orifice.

A 72-year-old man, previously diagnosed with SIT (**Figure 1a**), presented to our hospital on July 1, 2025, for “ Cough and expectoration for 3 months, shortness of breath for 1 month”.

### 2.2 History of past illness and family

The patient’s past medical history was significant for chronic obstructive pulmonary disease (COPD) and was a heavy smoker with a 54-year history, totaling 300 pack-years. His family history was significant for his father’s premature death due to heart disease and his sister’s death from liver cancer.

### 2.3 Physical, laboratory, imaging, and pathological examinations

Upon admission, vital signs were normal. His Karnofsky Performance Status (KPS) score was 80, and his body mass index (BMI) was 22.4 kg/m². Physical examination revealed a right-sided apical impulse. Dry rales were audible on auscultation of the left lung. Multiple non-tender lymph nodes of varying sizes (largest: 3.0 × 2.5 cm) were palpable in the left cervical and left inguinal regions.

Laboratory investigations revealed elevations in several tumor markers: pro-gastrin-releasing peptide (ProGRP) at 5000.00 pg/mL, cytokeratin 19 fragment (CYFRA 21-1) at 9.10 ng/mL, and carcinoembryonic antigen (CEA) at 89.50 ng/mL. Other tests, including arterial blood gas analysis, liver and renal function panels, complete blood count, coagulation profile, myocardial enzyme spectrum, and troponin, were all within normal limits.

Contrast-enhanced chest CT demonstrated a left hilar mass, highly suspicious for malignancy, causing obstructive atelectasis of the left lower lobe, along with enlarged mediastinal and left hilar lymph nodes suggestive of regional metastasis. Furthermore, ultrasonography identified hypoechoic masses, likely metastatic lymph nodes, in the left cervical and inguinal regions, with an additional suspicious lesion in zone III of the left neck (**Figures 1b-d**). Bronchoscopy confirmed a neoplasm at the left lower lobe bronchial orifice, which was biopsied (**Figure 1e**). Notably, while whole-body bone scintigraphy revealed a focal area of increased metabolic activity in the right forearm (recommended for periodic follow-up). contrast-enhanced magnetic resonance imaging (MRI) of the brain showed no abnormalities. To further characterize the lymphadenopathy, the suspicious cervical and inguinal lymph nodes were subjected to fine-needle aspiration (FNA).

**Figure 2:**
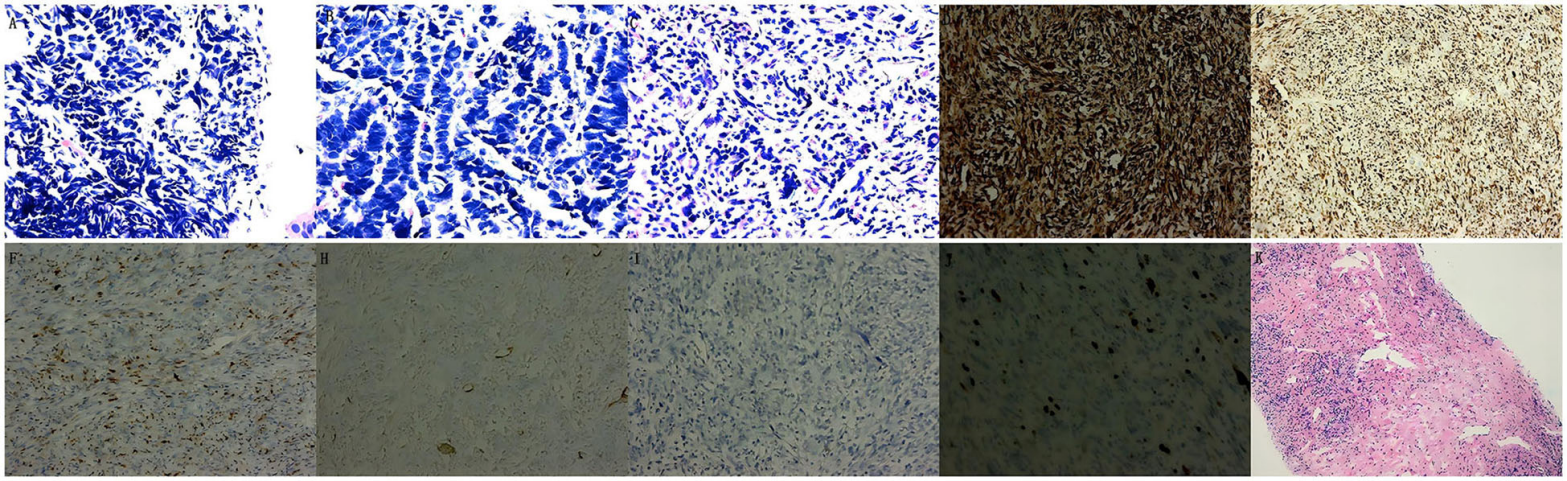
Histopathological and IHC results of the biopsy. Histopathological: **(a)** Depicts that the pathology of the lower lobe of the left lung indicates SCLC (HE, x400); **(b)** Depicts that the pathology of inguinal lymph nodes indicates mSCLC (HE, x400); **(c)** Depicts that the pathology of the left neck (zone III) indicates LGMS (HE, x400). IHC-Postoperative: **(d)** Vimentin (+); **(e)** Desmin (+); **(f)** S-100 (focal +); **(h)** SMA (-); **(i)** CK (-); **(j)** Ki-67 (2%). **(k)** Follow-up biopsy of the previously involved inguinal lymph node (November 28) showed no residual carcinoma.

The biopsy from the left lower lobe bronchus was diagnostic of SCLC (**Figure 2a**), supported by the immunohistochemical (IHC) staining results: CD56 (+), Syn (+), CgA (weakly +), cytokeratin (CK) (paranuclear dot +), with a high Ki-67 index (80%+) and PD-L1 TPS of ~10%; stains for P40 and CK5/6 were negative. Subsequent evaluation of the inguinal lymph node confirmed metastatic SCLC. In contrast, the cervical lymph node initially suggested a soft tissue sarcoma, raising the possibility of a second primary malignancy. To resolve this discrepancy, repeat core needle biopsies were performed. Final repeat biopsies confirmed metastatic SCLC in the inguinal node (CK+, CD56+, Syn+, TTF-1+, Ki-67 90%; Vimentin-) and identified the cervical node lesion as a low-grade myofibroblastic sarcoma (LGMS) (**Figures 2b, c**). The LGMS exhibited an immunoprofile positive for Vimentin, Desmin, focal Actin, CD56, focal CD99, and BCL-2, with a low Ki-67 index (2%), while being negative for CK, S-100, and CD34.

### 2.4 Final diagnosis

The final diagnosis was synchronous primary malignancies: a cervical LGMS and extensive-stage small cell lung carcinoma (ES-SCLC) with metastatic involvement of cervical and inguinal lymph nodes, staged according to the Veterans Administration Lung Study Group (VALG) criteria.

### 2.5 Treatment

A sequential treatment strategy was implemented. Treatment was initiated in July 2025 with two cycles of induction chemoimmunotherapy consisting of the EP regimen (etoposide 100 mg/m² on days 1-3; cisplatin 25 mg/m² on days 1-3) plus tislelizumab (200 mg), administered every three weeks. This regimen led to the complete resolution of metastatic lymphadenopathy in the cervical and inguinal regions, with the exception of the primary LGMS lesion. Subsequently, in August 2025, surgical resection of the cervical LGMS was performed by a senior professor of head and neck surgery. Postoperative pathology reconfirmed the diagnosis of LGMS (ICH: Vimentin (+) (**Figure 2d**), Desmin (+) (**Figure 2e**), S-100 (Scattered +) (**Figure 2f**), CD56 (-), CD99 (Weakly +), BCL-2 (+), CK (-) (**Figure 2i**), CD34 (-), Ki-67 (2%) (**Figure 2j**), CD68 (+), SMA (-) (**Figure 2h**), ALK (-)). Following surgery, the patient received four cycles of consolidation chemoimmunotherapy with the same EP regimen combined with tislelizumab. Upon completion of this phase, treatment continued with sequential thoracic radiotherapy, followed by maintenance immunotherapy with tislelizumab (200 mg every 3 weeks).

### 2.6 Efficacy and adverse events

**Figure 3:**
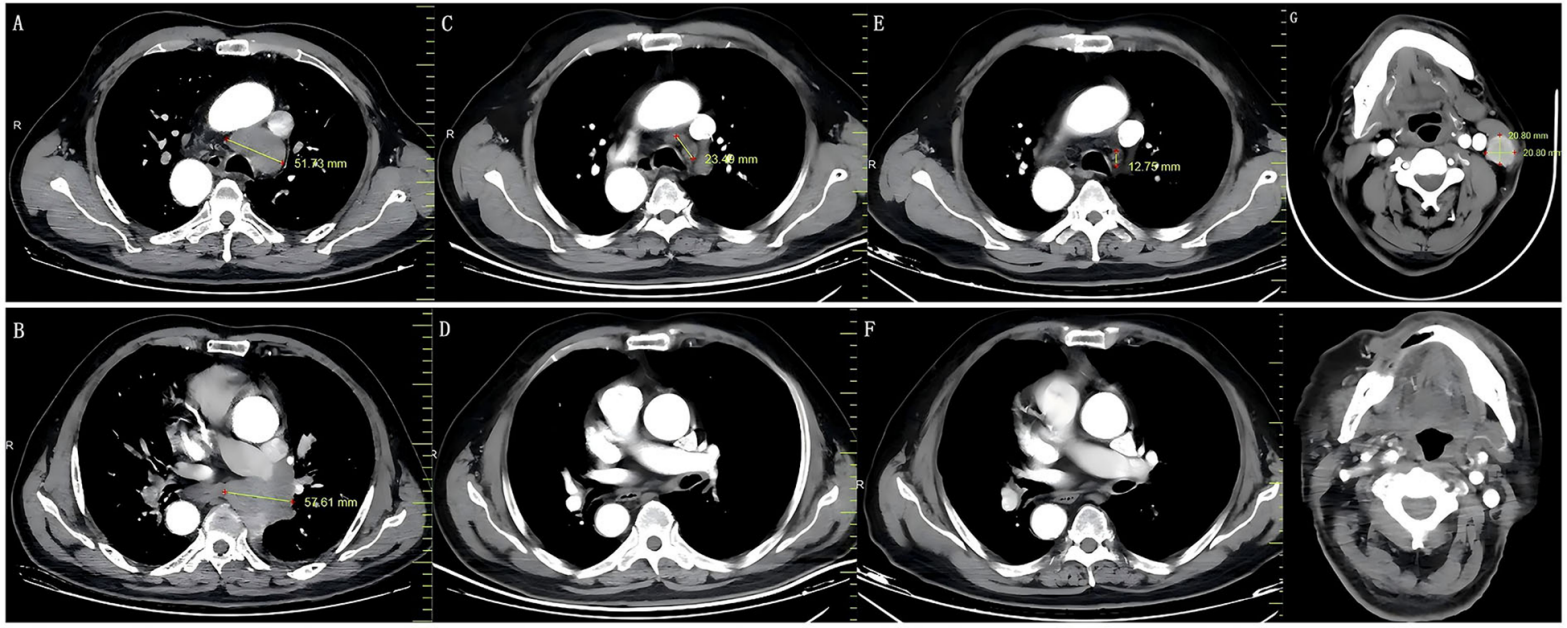
Therapeutic response assessment on chest enhanced CT. **(a, b)** Baseline imaging (July 1, 2025) of mediastinal lymph nodes and left hilar masses; **(c, d)** PR observed in the mediastinal lymph nodes and left hilar masses after chemoimmunotherapy (August 27, 2025); **(e, f)** CR achieved in the same regions after continued treatment (November 24, 2025); **(g, h)** The preoperative and postoperative imaging features of the left cervical LGMS.

After six cycles of chemoimmunotherapy and surgery, a follow-up core needle biopsy of the previously involved inguinal lymph node (November 28) showed no residual carcinoma (**Figure 2k**). According to the Response Evaluation Criteria in Solid Tumors (RECIST), version 1.1 (29), the patient achieved a clinical complete response (**Figures 3a–h**). Treatment-related adverse events included chronic kidney injury (serum creatinine fluctuating between 109.0 and 156.05 μmol/L) and grade IV neutropenia (nadir: 0.34 × 10^9^/L), graded according to the Common Terminology Criteria for Adverse Events (CTCAE) version 5.0 (30).

### 2.7 Follow-up

As of the last follow-up on February 14, 2026, the patient continued to receive maintenance immunotherapy with tislelizumab. No significant progression was observed in the cervical LGMS, thoracic SCLC, or inguinal lymph nodes, and overall progression-free survival (PFS) reached 7.5 months. However, serum levels of ProGRP and CEA, after an initial rapid decline during the first two treatment cycles, showed a progressive upward trend on subsequent evaluations, potentially indicating the development of rapid chemoresistance in SCLC. The follow-up period was relatively short (7.5 months), which was still insufficient to fully assess the recurrence of cervical LGMS. To enable early detection of local recurrence, it is recommended to perform enhanced chest and abdominal CT in conjunction with cervical MRI every 8 to 12 weeks during follow-up.

## 3 Discussion

### 3.1 Current landscape of LGMS

LGMS was initially reported by Vasudev et al. in 1978, although the detailed clinicopathological context of the lesions was not provided (31). In 1998, Mentzel et al. (32) confirmed this tumor by analyzing the immunohistochemistry and ultrastructure of 18 cases and named it “low-grade myofibroblastic sarcoma”. Montgomery et al. delineated its key clinical and pathological features through a review of 15 cases in 2001 (33). LGMS was classified as a separate solid tumor in 2020 (14). LGMS is a rare soft tissue sarcoma, with approximately 51.5% occurring in the head and neck region (11). It is a malignant mesenchymal neoplasm with myofibroblastic differentiation. Myofibroblasts are spindle cells derived from mesenchymal precursors that exhibit hybrid characteristics of both smooth muscle and fibroblastic lineages (34). Histopathologically, LGMS is characterized by diffusely infiltrating spindle cells arranged in bundles within a hyalinized collagenous stroma. While cellular atypia is observed, mitotic figures are typically scarce (35). Neoplastic cells display cytological and ultrastructural features consistent with myofibroblasts. The immunophenotype is heterogeneous but generally shows positivity for at least one myogenic marker (e.g., Desmin, α-SMA, or Calponin) and mesenchymal origin marker (vimentin) (36). In the current case, the tumor was immunoreactive for Vimentin, Desmin, and Actin, while being negative for CK. S-100 was negative in the biopsy specimen but showed scattered positivity in the resected tumor. Although this immunoprofile is not typical, such aberrant S-100 expression is recognized in a subset of sarcomas and does not rule out the diagnosis of low-grade myxoid sarcoma (LGMS), particularly when the morphological features and a broader myogenic immunohistochemical panel (Desmin+, Vimentin+) are supportive.

### 3.2 Diagnosis challenge in LGMS

LGMS is notably difficult to diagnose, primarily because it lacks characteristic clinical manifestations, distinct biological markers and imaging characteristics. LGMS is often misdiagnosed as leiomyosarcoma, fibrosarcoma, fibromatosis, nodular fasciitis, synovial sarcoma, or inflammatory myofibroblastic tumor (IMT) (35) (**Table 1**). Leiomyosarcoma is typically characterized by eosinophilic cytoplasm, centrally located blunt to round nuclei, and diffuse immunoreactivity for h-caldesmon—a feature absent in LGMS. In contrast, fibrosarcoma displays normochromic nuclei and pale cytoplasm, with tumor cells arranged in solid nodules, interlacing fascicles, or a fishbone-like arrangement. Vimentin is only focally expressed. The tumor cells in fibromatosis lack atypia or mitotic activity, are arranged in long fascicles or wavy patterns, and do not form interwoven fascicles or a fishbone arrangement. They demonstrate nuclear β−catenin expression, whereas most cells are negative for focal actin and/or desmin. The clinical features of nodular fasciitis are characterized by rapid growth (in < 3 months), nodule size <3 cm, occurrence predominantly in patients under 30 years of age, and does not infiltrate muscle or bone. Histologically, it shows tissue clefts, and Pathological mitotic figures may be absent, which lacks nuclear atypia and necrosis. Synovial sarcoma displays biphasic epithelial and mesenchymal differentiation with fascicular spindle cells, and is typically positive for CK, epithelial membrane antigen (EMA), and CD99, which is distinguish it from LGMS. IMT exhibits a mixed cellular composition with myxoid and vascular elements, The tumor was also positive for ALK and CK. Although histopathological examination remains the diagnostic gold standard for tumor classification, the diagnosis of LGMS in this case was confirmed through an comprehensive approach incorporating clinical presentation, imaging findings, and a broad immunohistochemical panel, in the absence of confirmatory molecular testing, which we acknowledge as a limitation.

**Table 1:** Histopathological and immunohistochemical features of LGMS and its differential diagnoses.

Tumor	Histopathological features	Immunohistochemical
LGMS	1. Spindle or stellate tumor cells arranged in interlacing fascicles. 2. Eosinophilic to amphophilic cytoplasm with fusiform nuclei, mild pleomorphism. 3. low mitotic rate (1–6/10 HPFs) 4. Abundant microfilaments, microtubules, and collagen fibers	Positivity: At least one myogenic marker (such as Desmin, α-SMA, Vimentin or Calponin), Negative: H-caldesmon, ALK, CK
Fibrosarcoma	1. Nuclei are normal, cytoplasm is pale 2. Distributed in solid nodules or sheets, interwoven bundles, or a fishbone-like arrangement	Positivity: Vimentin (focally +) Negative: α-actin (-), Desmin (-)
Fibromatosis	1. Arranged in long fascicles or waves, without interwoven fascicles 2. Mitotic figures are visible, no atypical nuclei	Positivity: Catenin (+), Negative: Actin (-), Desmin (-)
Leiomyosarcoma	1. Atypical cellular morphology, rounded nuclei with occasional vacuoles 2. Eosinophilic cytoplasm, central nuclei are blunt and round	Positive: H-caldesmon (+), a-actin (+) Negative: Specific markers for other lineages (e.g., S-100, CK)
Nodular Fasciitis	1. Comprises hyperplastic, obese, and uniform spindle-shaped cells with a myxomatous 2. Inflammatory cell infiltration 3. Irregular tissue clefts, No pathological mitotic figures	–
Synovial Sarcoma	1. Monophasic: Uniform spindle cells in fascicles. 2. Biphasic: Epithelial glands or nests within spindle cell stroma.	Positive: Cytokeratin (+), EMA (+), CD99 (+), Vimentin (+). Negative: Specific myogenic markers (Desmin, h-caldesmon)
IMT	Distinguished by a well- demarcated and mixed-cell composition with myxoid and vascular features	Positive: ALK (+), CK (+), α-SMA, Vimentin. Negative: Desmin (variable), S-100.

### 3.3 Treatment challenges in LGMS with lymph node metastasis of SCLC

There is no established consensus on the optimal treatment regimen for LGMS. Based on available evidence from reported cases, wide surgical excision with R0 margins constitutes the primary therapeutic approach (37). After surgical removal, most patients with LGMS have a good prognosis, but there are still some cases showing recurrence. The recurrence rate of low-grade myofibroblastic sarcoma (LGMS) in the head and neck region ranges from 25% to 40%, with the highest risk observed for tumors located in the nasal cavity or paranasal sinuses (38). Some studies (32, 33, 39) suggest that radiotherapy or chemotherapy combined with surgery can prevent recurrence. However, there are also reports suggesting that LGMS is not sensitive to postoperative radiotherapy and chemotherapy (40, 41). A study of 96 patients by Xu et al. (42) demonstrated that chemotherapy and radiation conferred limited survival benefit in LGMS, particularly among those with negative surgical margins. Therefore, the effects of postoperative radiotherapy and chemotherapy still need to be further studied and determined. To our knowledge, there are no reported cases of LGMS in the neck combined with cervical lymph node metastasis of SCLC, nor are there any recommended treatment methods. The different biological behaviors of SCLC (aggressive, systemic) and LGMS (indolent, localized) have created a therapeutic conflict. Given the marked chemosensitivity and rapid tumor regression typical of SCLC, in contrast to the biology of LGMS, the multidisciplinary team (MDT) recommended a sequential strategy of chemotherapy followed by surgery. The patient received two cycles of the EP regimen combined with an immune checkpoint inhibitor, after which LGMS resection was performed. This case demonstrates the effectiveness of a sequential treatment approach tailored to the distinct biological characteristics of each tumor type.

### 3.4 Management challenges in SCLC with SIT

SIT is a rare autosomal recessive congenital defect (43), characterized by a mirror-image reversal of visceral anatomy. This condition is also associated with variations in the branching patterns of pulmonary vessels and bronchi. To our knowledge, the co-occurrence of SIT with SCLC has not been previously reported in China. Chen et al. (5) summarized three cases of SCLC combined with SIT, all of which received conservative management. Among SIT patients, approximately 20-25% of them presented with Kartagener syndrome, a disorder defined by primary ciliary dyskinesia (PCD) (2). The present case was not complicated by Kartagener syndrome; isolated SIT is often asymptomatic. Notably, the anatomical dislocation of key thoracic structures - including the interlobar fissure, bronchial tree, and pulmonary vessels - throughout the clinical management may complicate the diagnosis, precise localization, and radiotherapy planning for SCLC in this setting. Nevertheless, this anatomical variance did not diminish the inherent sensitivity of SCLC to systemic treatment.

As a high-grade neuroendocrine carcinoma strongly linked to smoking (21). SCLC exhibits rapid tumor kinetics, a high proliferative index, and a pronounced tendency for early, widespread metastasis, all contributing to its exceptionally poor prognosis (44). Globally, SCLC accounts for an estimated 250,000 new cases annually (21). Approximately 70% of patients present with extensive-stage disease (ES-SCLC) at diagnosis (45), and the overall 5-year survival rate remains below 7% (46).

The advent of programmed cell death 1 (PD-1) or ligand 1 (PD-L1) inhibitors has significantly improved OS and PFS in patients with ES-SCLC, establishing chemoimmunotherapy as the current first-line standard. Multiple large-scale phase III trials have demonstrated that adding a PD-L1 inhibitor [atezolizumab (47), durvalumab (48), Adebrelimab (49), or Benmelstobart (50)] to the EP/EC chemotherapy regimen significantly improves outcomes over chemotherapy alone. The reported median overall survival (OS) with the combination ranges from 12.3 to 19.3 months versus 10.3 to 11.9 months with chemotherapy alone.

The efficacy of PD-1 inhibitors in ES-SCLC shows considerable heterogeneity. While pembrolizumab (51) and nivolumab (52) improved PFS without translating into a significant OS benefit in their respective trials, other agents in combination with chemotherapy have demonstrated clear OS advantages. The RATIONALE-312 trial demonstrated that tislelizumab [a PD-1 inhibitor) plus chemotherapy significantly improved OS (53), with a mOS of 15.5 months (95% CI 13.5–17.1). Furthermore, confirmatory phase III trials showed that serplulimab (mOS 15.8 months in ASTRUM-005 (54)] and toripalimab [mOS 14.6 months in EXTENTORCH (55)] also significantly outperformed chemotherapy alone. In the MAURIS trial, receipt of ≥5 cycles of chemotherapy was associated with superior clinical outcomes compared to only 4 cycles (56). Guided by the RATIONALE-312 and MAURIS study regimen, the patient was treated with six cycles of tislelizumab in combination with EP chemotherapy, and maintenance therapies with tislelizumab, which resulted in a complete pathological response. The observed rise in ProGRP following its initial decrease serves as an early indicator of potential disease recurrence in this patient. This observation aligns with the well-documented pattern of early disease progression in most ES-SCLC patients within 3–4 months of maintenance therapy initiation (57). Both the Chinese (2025 edition) and ASTRO guidelines recommend consolidative thoracic radiotherapy for ES-SCLC patients who achieve a complete or partial response (CR/PR) to initial chemoimmunotherapy (58). Therefore, this patient received thoracic radiotherapy (TRT) at fractionated doses of 55 Gy/25f with 2.2 Gy per fraction, no significant adverse reactions were observed. Given the absence of prior case reports on thoracic radiotherapy for SCLC with SIT, the treatment was strictly planned and delivered according to the Chinese radiotherapy guidelines for SCLC by two senior radiation oncologists. Simulation was performed using contrast-enhanced CT with a slice thickness of ≤5 mm. Daily cone-beam CT (CBCT) was used for image-guided radiotherapy (IGRT) before every fraction to ensure accurate targeting. For intensity-modulated radiotherapy (IMRT), the gross tumor volume (GTV) included the post-chemotherapy residual primary lesion and the pre-chemotherapy positive mediastinal lymph nodes. A planning target volume (PTV) was generated by adding a 5–8 mm margin to the GTV. Dose-volume constraints were adhered to as follows: for lungs, V20 < 23%, V30 < 13%, and mean dose < 13 Gy; for the spinal cord planning organ at risk volume (PRV), Dmax < 40 Gy; for the heart, mean dose < 20 Gy and V30 < 30%.

## 4 Conclusions

We report the first case of synchronous LGMS and SCLC in a patient with SIT, highlighting the diagnostic and therapeutic complexities of managing multiple rare diseases. A initial favorable response was achieved through a multimodal approach integrating surgery, chemotherapy, immunotherapy, and radiotherapy. The treatment sequence was strategically tailored: systemic therapy was prioritized to target the highly chemosensitive SCLC and shrink metastatic lymph nodes, followed by surgical resection of the second primary tumor (LGMS). Furthermore, despite the potential challenges posed by anatomical variations (e.g., in the interlobar fissure, bronchial tree, and pulmonary vessels), thoracic radiotherapy was safely and effectively delivered following meticulous anatomical evaluation by an experienced radiation oncology team. We present this case as a initial treatment-planning report for this sequential strategy, acknowledging that longer follow-up is required to confirm the durability of the response and to monitor for late recurrence of LGMS or relapse of SCLC.

## Data Availability

The original contributions presented in the study are included in the article/supplementary material. Further inquiries can be directed to the corresponding author/s.
